# Emergence of two novel viruses in Tibetan pigs with porcine diarrheal disease on the Tibetan plateau of southwest China

**DOI:** 10.3389/fvets.2025.1654388

**Published:** 2025-09-03

**Authors:** Haohao Lu, Min Zhang, Danjiao Yang, Xue Gao, Shuo Feng, Yiwen Pei, Zhidong Zhang, Long Zhou

**Affiliations:** ^1^Key Laboratory of Veterinary Medicine in Universities of Sichuan Province, College of Animal Science and Veterinary Medicine, Southwest Minzu University, Chengdu, China; ^2^Institute of Animal Science of Ganzi Tibetan Autonomous Prefecture of Sichuan Province, Kangding, China; ^3^Key Laboratory of Ministry of Education and Sichuan Province for Qinghai-Tibetan Plateau Animal Genetic Resource Reservation and Utilization, Chengdu, China

**Keywords:** dicistrovirus, St-Valerien-like virus, Tibetan pig, porcine diarrheal disease, phylogenetic analysis

## Abstract

Porcine diarrheal disease is a major cause of morbidity in Tibetan piglets, however, the causative agents of this disease are rarely reported. Here, the viral diversity associated with porcine diarrheal disease was investigated by analyzing the viral communities from Tibetan pigs on the Tibetan plateau of southwest China. The results revealed that 13 mammalian viruses were identified in a pooled sample. Interestingly, it is the first time that dicistrovirus (DCV) was discovered in pigs and St-Valerien-like virus (StVV) was identified in China. Furthermore, the complete genome sequence of the two strains (DCV/porcine/CHN/SCdc-2024 and StVV/porcine/CHN/SCdc-202402) were obtained. Sequence comparisons and phylogenetic analysis showed that the swine-origin DCV/porcine/CHN/SCdc-2024 strain was classified into the family *Dicistroviridae* with an unassigned genus, and showed distant relationship with other dicistrovirus strains in established genera, may represent members of a potential new genera within the *Dicistroviridae* family. Additionally, the novel StVV strain StVV/porcine/CHN/SCdc-202402 was classified into the *Valovirus*, whereas showed a unique phylogenetic branch compared with other swine-origin StVV strains. Notably, further case–control investigation in the 87 fecal samples using specific RT-PCR found a high DCV-positive detection rate (77.8%) in diarrheic samples with a significant *p* value (< 0.0001), suggesting the DCV might associated with diarrhea in pigs. Our study reports for the first time the emergence of DCV in pigs and StVV in China, highlighting the need for further research on pathogenicity and transmission in swine hosts.

## 1 Introduction

The Tibetan pig is a geographically isolated pig breed that is found on the Qinghai-Tibetan plateau of China. The animals were grow in the natural and harsh environment with an average altitude of more than 3,000 m (1). They have adapted to the adverse climatic conditions of Qinghai-Tibet plateau and show significant phenotypic and physiological differences from domestic pigs from low elevation areas (2). Tibetan pig farming is an important source of meat and economy of the nomads. Viral diarrheal disease is a major health problem in piglets worldwide causing tremendous economic losses to the swine industry worldwide, such as porcine epidemic diarrhea (PED) (3), transmissible gastroenteritis (TGE) (4), rotavirus infection (5), and porcine deltacoronavirus infection (6). Due to the remote geographical location and high altitude of Tibetan pig farming, there is limited studies on viral diseases in these animals. Recently, the high-throughput sequencing has been successful for analyzing the viromes in Tibetan pigs with diarrheal disease, and several new viruses have been identified, such as bufavirus, rabovirus, and pasivirus (7), however, the dicistrovirus and St-Valerien-like virus have never been reported in these animals.

The *Dicistroviridae* is a family of small non-enveloped, positive-sense, non-segmented RNA viruses, and is classified in the order *Picornavirales* (8). Dicistroviruses (DCVs) have been detected in many invertebrate hosts worldwide, such as aphids (9), leafhoppers (10), crickets (11), flies (12), bees (13), ants (14), silkworms (15), crabs (16), and shrimps (17). Some of them have significant economic impact on their hosts, such as honey bees and shrimps (17, 18). Recent studies reported that DCVs existed in fecal samples of vertebrate hosts, including geese (19), bats (20), squirrels (21), birds (22), and pandas (23). Moreover, the DCV RNA could be detected in stool samples from Australian children with acute diarrhea (24). However, there are no information on the epidemiology of DCV in livestock.

The *Caliciviridae* is a family of small non-enveloped, positive-polarity, non-enveloped RNA viruses, and is classified in the order *Picornavirales* (25). Caliciviruses are a major cause of diarrhea in pigs and have been classified into 3 established genera: *Valovirus*, *Sapovirus*, and *Vesivirus* (26–28). Moreover, newly recognized porcine caliciviruses, St-Valerien-like viruses (StVV), which belong to genus *Valovirus*, were first identified in pig feces from Canadian swine farms during 2005–2007 (29). Subsequently, StVV strains were detected in swine in Italy, U.S.A., and Japan from 2008 to 2013 (28, 30, 31). To date, the prevalence and genetic diversity of StVV in other countries are unknown.

In this study, we report the genome structure and phylogenetic properties of two novel viruses (DCV and StVV) identified in fecal samples of Tibetan pigs associated with porcine diarrheal disease in China. To our knowledge, this is the first time that DCV was discovered in pigs and StVV was identified in China.

## 2 Materials and methods

### 2.1 Sample collection and preparation

In 2024, a total of 87 fresh fecal samples, including 60 healthy (asymptomatic) samples and 27 diarrhea samples, were collected from 10- to 30-day-old individual Tibetan piglets. These samples were obtained from a pig farm in Daocheng County, Ganzi Tibetan autonomous prefecture of Sichuan province, with an average altitude of ~4,000 m above sea level. These specimens were taken using throwaway tools, stored on dry ice, and brought immediately to the laboratory. The stool samples were vortexed vigorously for 10 min after resuspending in 5 mL of Dulbecco’s Modified Eagle Medium (DMEM); 1 mL of supernatant was taken from each piglet fecal sample and combined into a pool, which was then vortexed and centrifuged. After passing the combined samples using 0.22 μm filters (Millipore, Burlington, MA, United States), the filtrated solution was concentrated using an ultracentrifuge (Beckman, United States) with 200,000 × *g* and 4°C for 2 h; the precipitation was then resuspended in 1 mL DMEM. Ultimately, TRIzol was used to extract RNA from concentrated solution samples for next-generation sequencing. Random hexamers and the Superscript III RT reverse transcriptase kit (Invitrogen, Carlsbad, CA, United States) were then used for reverse transcription.

### 2.2 Viral metagenomic sequencing

A library was constructed, and Illumina sequencing was carried out at Shanghai Tanpu Biotechnology Co., Ltd. (Shanghai, China). The resulting cDNA fragments were joined at both ends with sequencing linkers, and after bridge PCR amplification, the cDNA library was sequenced using the paired 150 bp sequencing technique on the Illumina Novaseq6000 platform. The raw reads were filtered and trimmed using FASTP^1^ to remove low-quality reads (reads < 50 bp and phred score <20) and sequencing adapters. The BBMAP tool performed read-mapping subtraction between ribosomal RNAs and host reads. SPAdes v3.14.1^2^ was used for *de novo* genome assembly. These acquired assembled scaffolds have the best BLAST hits to the NCBI nucleotide database and restricted the minimum contig length to 100 bases. Finally, Kraken2 (v 2.0.8-beta) classified high-quality filtered reads by precisely aligning k-mers (17-mer) of varying lengths.

### 2.3 Bioinformatic analyses

The NCBI ORFfinder^3^ function was used to predict potential ORFs. The NCBI conserved domain database^4^ is used to search for conserved domains. The MegAlign tool (DNASTAR, Madison, WI, United States) was used to analyze the nucleotide (nt) and inferred amino acid (aa) sequences. To infer phylogenetic relationships, multiple sequence alignment and phylogenetic analysis were carried out using the MEGA 11 software. Maximum likelihood (ML) phylogenetic trees were constructed using the complete genomes of isolates in this study with a Tamura-Nei nucleotide substitution model. 26 and 27 representative reference strains belonging to different viral genera (or unassigned groups) within the family *Dicistroviridae* and *Caliciviridae* were obtained from the ICTV^5^^,^ ^6^ and used to reconstruct the phylogenetic tree of DCV and StVV, respectively. The bootstrap values were set to 1,000.

### 2.4 DCV and StVV screened using RT-PCR

The prevalence of DCV and StVV in pigs was further investigated by screening the 87 stool samples from Tibetan pigs. Viral RNAs were extracted from stool samples and reverse transcribed. Two pair of PCR detection primers targeting the DCV genome was designed, using Primer5.0 software, based on virus metagenomics sequencing data. The detection primer sequence of DCV was: forward 5’-AACGAGAACGGTTATGAT-3′, reverse 5’-AAGTGACGGATTGACAGA-3′, and the amplified fragment 681 bp; the detection primer sequence of StVV was: forward 5’-CATCGGTGCCTATGAAGA-3′, reverse 5’-AGCGGAGTTTGAGGAGAA-3′, and the amplified fragment 824 bp. Detection of DCV and StVV used high-fidelity polymerase Platinum SuperFi II DNA (Invitrogen, United States) with the following PCR program: 94°C for 3 min, 30 cycles of 94°C for 30 s, 55°C for 30 s, and 72°C for 60 s; and final extending at 72°C for 10 min. A significantly different rate of viral detection was calculated using Fisher’s exact test. To confirm the genomic sequences of the two novel viruses, five and six pairs of overlap primers were designed to amplify the whole genomes of DCV and StVV, respectively (Supplementary Table S1). The amplified PCR products were cloned into the pMD19-T vector (TaKaRa, Dalian, China) and sequenced by the commercial service (Sangon, Shanghai, China) using Sanger sequencing.

## 3 Results and discussion

### 3.1 Overview of virome in the pooled diarrhea samples

**Figure 1:**
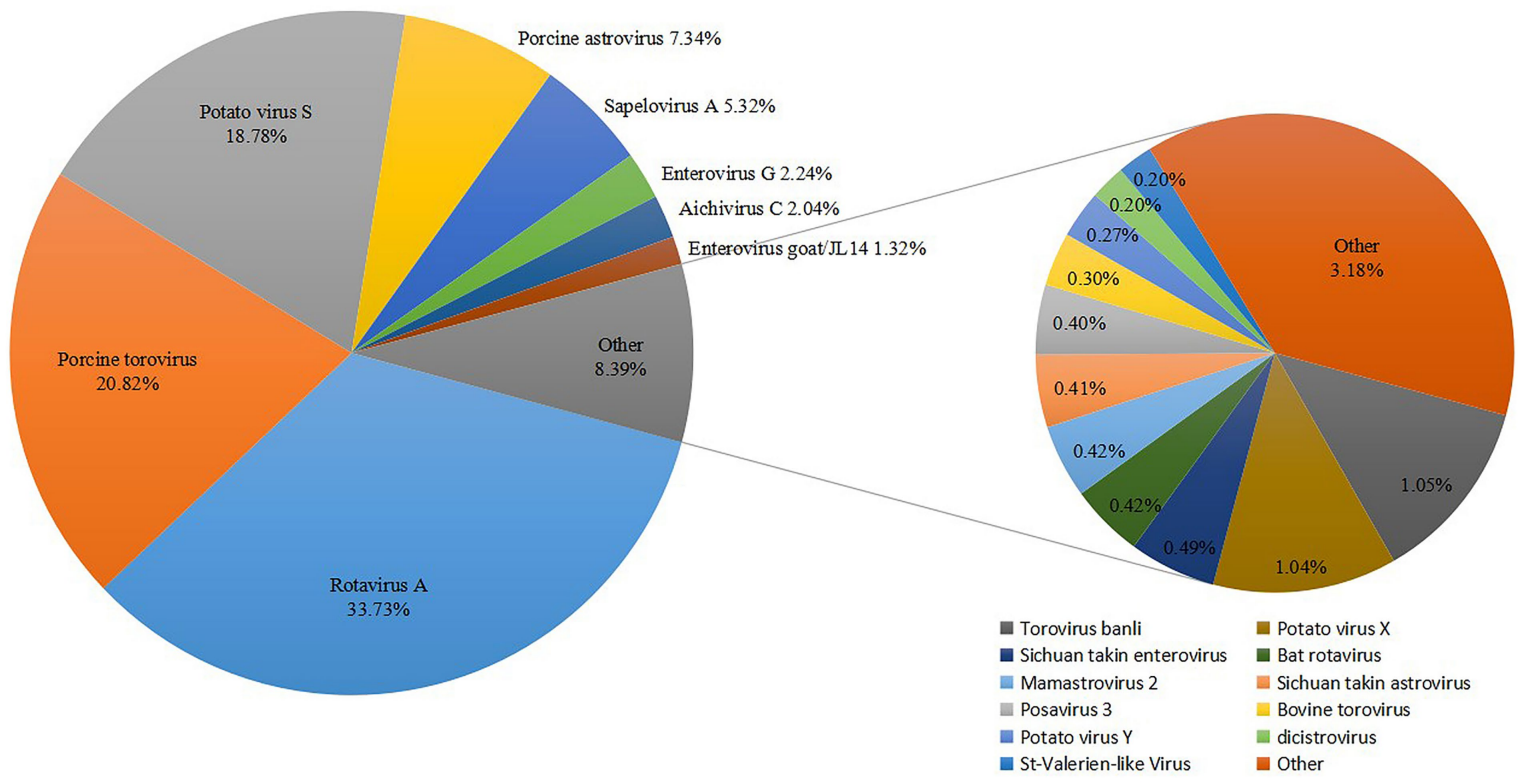
Classification and percentage of virus sequences detected from diarrhoeic fecal samples from Tibetan piglets.

Overall, metagenomic analysis of 27 diarrhea samples yielded 21,013,876 reads annotated to mammalian viruses. Using BLASTx software (E-value < 10^−5^), members of 13 different mammalian-associated viruses were identified, which, in order of sequence read abundance, were as follows: Rotavirus A (33.73%), porcine torovirus (20.82%), porcine astrovirus (7.34%), sapelovirus A (5.32%), enterovirus G (2.24%), aichivirus C (2.04%), enterovirus goat/JL14 (1.32%), bat rotavirus (0.42%), mamastrovirus 2 (0.42%), posavirus 3 (0.4%), bovine torovirus (0.3%), dicistrovirus (0.2%), St-Valerien-like virus (0.2%) (Figure 1). Six viruses in the study were previously reported to be associated with animal diarrheal disease, including porcine rotavirus (5), torovirus (32), sapelovirus (33), astrovirus (34), enterovirus (35) and aichivirus (36). Strikingly, enterovirus goat/JL14 strain was detected in goats with severe watery diarrhea in 2014 (37), and bovine torovirus was frequently detected in cattle (38), suggesting potential interspecies transmission of enterovirus among Tibetan pigs and other livestock. This finding agrees with our previous reports (7). Notably, the sequence reads of bat rotavirus indicate a risk of cross-species transmission between Tibetan pigs and wild animals (39). Interestingly, it is the first time of detecting DCV in pigs and StVV in China.

### 3.2 Genome sequence analysis of DCV/porcine/CHN/SCdc-2024

**Figure 2:**
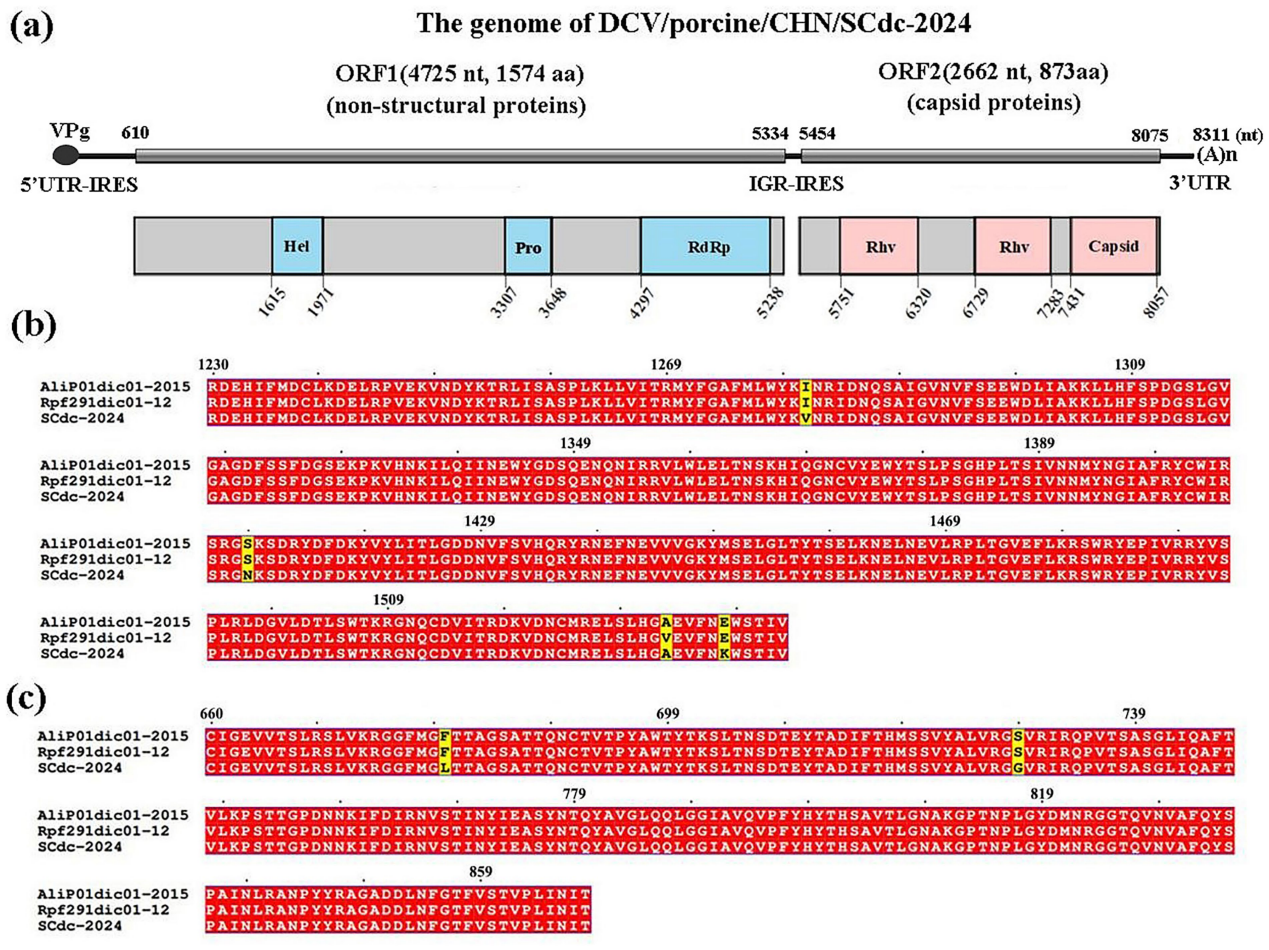
Schematic representation of the putative genomic organization and phylogenetic tree based on full-length genomic sequence of DCV/porcine/CHN/SCdc-2024. **(a)** Hel, RNA helicase; Pro, 3C cysteine protease; RdRP, RNA-dependent RNA polymerase; Rhv, rhinovirus capsid protein-like; capsid, cricket paralysis virus (CrPV) capsid protein-like. **(b)** Location of the mutation site of the DCV/porcine/CHN/SCdc-2024 strain in the RdRp. The unique amino acids are indicated with highlighted boxes. **(c)** Location of the mutation site of the DCV/porcine/CHN/SCdc-2024 strain in the capsid. The unique amino acids are indicated with highlighted boxes.

The genomes of *Dicistroviridae* are approximately 8.5–10.0 kilobases in length and contain two non-overlapping open reading frames (ORFs), including ORF1 and ORF2 (40). In the present study, the complete nucleotide sequence of a DCV strain was determined by RT-PCR from positive samples and the virus was named DCV/porcine/CHN/SCdc-2024 (GenBank Accession No. PQ817971). The genome length of DCV/porcine/CHN/SCdc-2024 was 8,311 bp, with 37.76% GC content. Potential ORFs were predicted using the NCBI ORFfinder and the Geneious ‘Find ORFs’ function. Two putative ORFs (ORF1 and 2) were identified within the genome of DCV/porcine/CHN/SCdc-2024. The 5′ proximal ORF1, starting at 610 nt and ending at 5,334 nt (4,725 nt long) encoded a predicted non-structural protein of 1,574 aa. The 3′ proximal ORF2 starts at 5454 nt and ends at 8,075 nt (2,622 nt long) encoded the structural protein of 873 aa. A search for conserved domains using the NCBI conserved domain database showed that the ORF1-encoded protein contains the RNA helicase (Hel), cysteine protease (Pro), and RNA-dependent RNA polymerase (RdRP) domains, while the ORF2-encoded two rhinovirus capsid protein-like (Rhv) and a cricket paralysis virus (CrPV) capsid protein-like (Figure 2a). In addition, a small genome-linked virus protein (VPg) is covalently attached to the 5′-end, and a polyadenylated tail is present in the 3′-terminus of the genome. Two distinct internal ribosome entry sites (IRES) have been identified in the 5′-untranslated region (UTR) and intergenic region (IGR) to translate the replicase in ORF1 protein and capsid protein in ORF2, respectively (Figure 2a). The results indicated that the DCV/porcine/CHN/SCdc-2024 strain has a typical genome structure of *Dicistroviridae* (8).

Amino acid mutation analysis showed that RdRp and Capsid are the most variable regions in the nonstructural proteins and structural proteins in the genome of DCV/porcine/CHN/SCdc-2024, respectively. In the novel strain, three unique amino acids, such as V81, N1409 and K1538, were identified in the *RdRp* gene (Figure 2b), and two unique amino acids, such as L680 and G729, were identified in the *Capsid* gene when compared to the two *Ailurus fulgens*-origin strains (Alip01dic01-2015 and Rpf191dic01-12) (Figure 2c).

**Figure 3:**
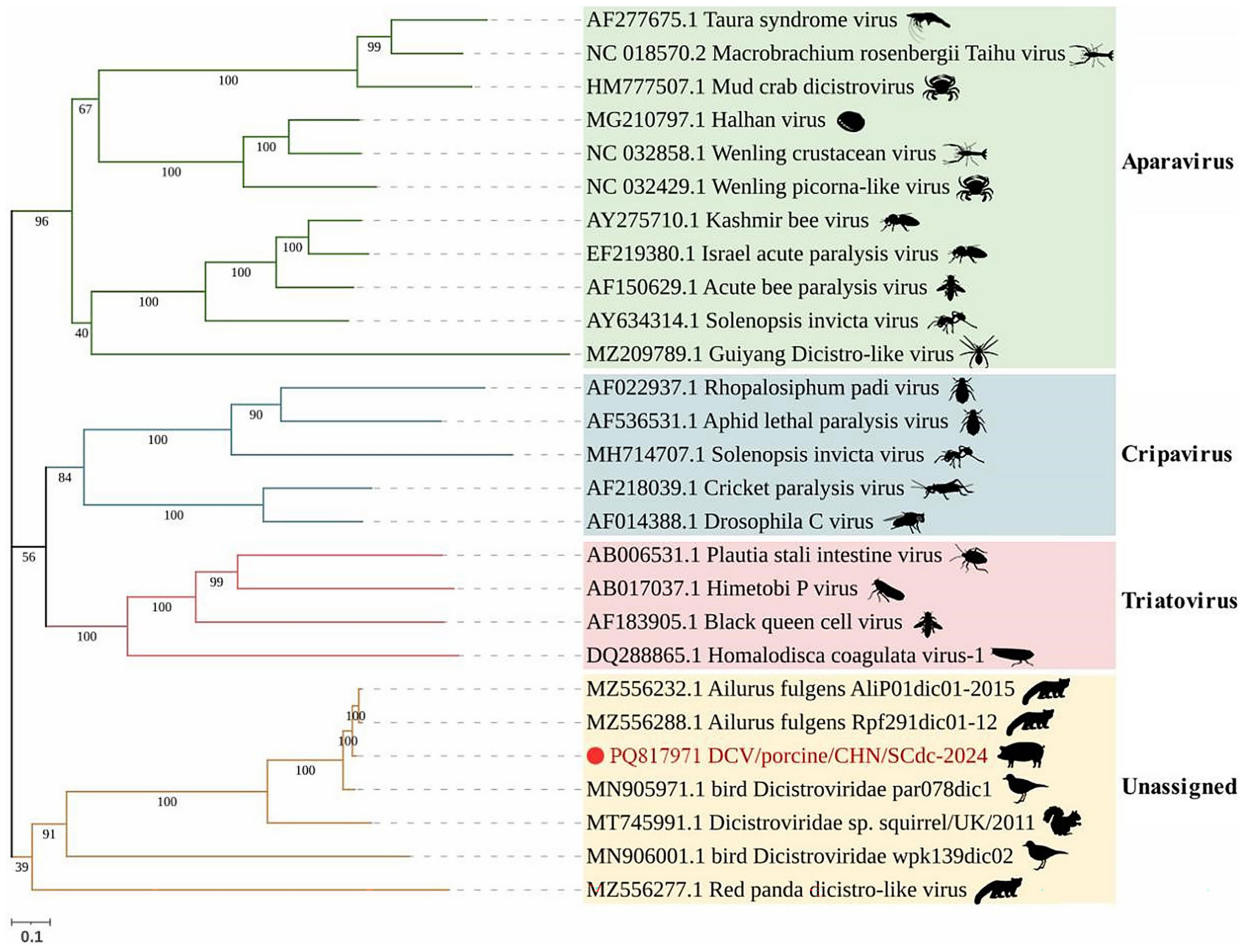
Schematic representation of the phylogenetic tree based on full-length genomic sequence of DCV/porcine/CHN/SCdc-2024. Maximum likelihood (ML) phylogenetic trees were inferred using a whole genome sequences, the phylogenetic trees were constructed with 1,000 replicates using the MEGA11 program. The scale bar indicates evolutionary distance in numbers of substitutions per amino acid site.

According to the latest ICTV, *Dicistroviridae* is composed of three established genera, including the *Apavirus* genus with 6 species (e.g., *apisacutum*, *cancerluti*, *israelense*, *kashmirense*, *tauraense*, and *vallesi*), the *Cripavirus* genus with 5 species (e.g., *drosophilae*, *grylli*, *mortiferum*, *porter*, and *ropadi*) and the *Triatovirus* genus with 5 species (e.g., *himetobi*, *hocoagulatae*, *nigereginacellulae*, *plastali*, and *triatomae*) (8). In phylogenetic analysis, all *Dicistroviridae* viruses are classified into four clades: *Aparavirus*, *Cripavirus*, *Triatovirus* and an unassigned clade. The majority of DCVs from arthropods were classified into the above three established genera with bootstrap values ranged from 40 to 100, 56 to 100, and 56 to 100, respectively, and the DCV strains identified in vertebrates, such as pandas (23, 41), birds (22), squirrels (21), were divided into a novel unassigned clade with bootstrap values ranged from 39 to 100. The strain DCV/porcine/CHN/SCdc-2024 from Tibetan pigs showed a distant genetic relationship with *Aparavirus*, *Cripavirus*, and *Triatovirus,* and was classified in the unassigned clade (Figure 3). The results revealed that the DCV strains originating from vertebrate animals might have formed a new evolutionary branch.

To examine the genetic variation, the nucleotide and deduced amino acid (aa) sequences of the ORF1 and ORF2 genes of this isolates, including 16 strains from *Triatovirus*, *Aparavirus*, and *Cripavirus*, and 4 reference strains from the unassigned group, were compared. The results showed that the genome of DCV/porcine/CHN/SCdc-2024 shared the highest nucleotide identity (97.4%) with a DCV strain from pandas (*Ailurus fulgens*), which was identified in fecal samples from pandas in 2015 (23). However, the genome of DCV/porcine/CHN/SCdc-2024 showed lower identity (< 40%), in terms of 28.6–36.3%, 29.8–31.7%, and 33.7–38.0%, compared to reference genomes from genus *Aparavirus*, *Cripavirus*, and *Triatovirus*, respectively. The ORF1 and ORF2 of DCV/porcine/CHN/SCdc-2024 shared 72.9–98.2% nucleotide (71.9–99.6% aa) sequence identity with unassigned strains, a significantly higher percentage than with the other reference strains from *Aparavirus* (21.3–52.6% nt, 7.6–47.6% aa), *Cripavirus* (29.0–48.2% nt, 25.7–42.6% aa), and *Triatovirus* (38.5–47.0% nt, 32.0–41.5% aa). In addition, the IGR IRES of SCdc-2024 shared 91.6–99.8% nucleotide identity with unassigned strains, which was higher than that with the other strains from *Aparavirus* (59.0–84.5%), *Cripavirus* (10.9–84.3%), and *Triatovirus* (80.3–84.0%) (Table 1). Although the formal genus demarcation criteria of *Dicistroviridae* from ICTV have not yet been established (8), different genera display unique sequence and topological characteristics in the IGR IRES and form separate groups on phylogenetic analysis, indicating that DCV/porcine/CHN/SCdc-2024 and other unassigned strains from vertebrate hosts may represent a new genus within the family *Dicistroviridae*.

**Table 1:** Nucleotide and amino acid sequence identity values for different regions compared with other *Dicistroviridae* reference strains.

Genus	Species	Accession no.	ORF1 (nt/aa)	ORF2 (nt/aa)	IRES (nt)	Complete genome (nt)
*Aparavirus*	*Penaeus vannamei*	AF277675.1	38.0/31.7	36.4/21.8	79.7	28.6
	Bee	AY275710.1	21.3/21.4	52.6/33.3	79.0	33.8
	*Apis mellifera*	AF150629.1	39.2/32.6	51.9/32.0	82.5	33.4
	*Scylla serrata*	HM777507.1	21.5/17.0	41.9/21.9	59.0	26.1
	Honeybee	EF219380.1	38.8/33.0	51.3/32.8	82.9	34.5
	*Solenopsis invicta*	AY634314.1	47.4/47.6	21.6/7.6	84.5	36.3
*Cripavirus*	*Drosophila melanogaster*	AF014388.1	48.2/38.0	44.3/34.0	82.9	29.8
	Aphid	AF022937.1	30.1/26.9	46.1/40.0	44.1	30.7
	*Teleogryllus oceanicus*	AF218039.1	46.9/37.3	46.4/34.2	81.6	27.9
	*Rhopalosiphum padi*	AF536531.1	29.0/25.7	44.6/40.2	84.3	31.7
	*Solenopsis invicta*	MH714707.1	30.9/28.4	42.4/42.6	10.9	31.5
*Triatovirus*	*Plautia stali*	AB006531.1	39.6/36.4	45.2/38.1	83.3	36.8
	*Laodelphax striatellus*	AB017037.1	40.1/38.9	47.0/36.5	84.0	33.7
	*Triatoma infestans*	AF178440.1	41.2/37.7	46.4/37.5	83.9	37.5
	*Apis mellifera*	AF183905.1	46.2/41.5	45.3/39.4	80.3	36.6
	Homalodisca coagulata	DQ288865.1	38.5/37.3	43.6/32.0	83.3	38.0
Unassigned	*Ailurus fulgens*	MZ556232.1	**97.1/98.8**	**98.2/99.6**	**99.8**	**97.4**
	*Ailurus fulgens*	MZ556288.1	**97.1**/98.6	98.0/99.4	99.7	96.8
	Bird	MN905971.1	94.6/98.8	96.4/99.6	99.3	94.9
	*Sciurus vulgaris*	MT745991.1	75.0/73.9	72.9/71.9	91.6	73.4

The highest nucleotide and amino acid identities are indicated in bold typeface.

### 3.3 Genome sequence analysis of StVV/porcine/CHN/SCdc-202402

**Figure 4:**
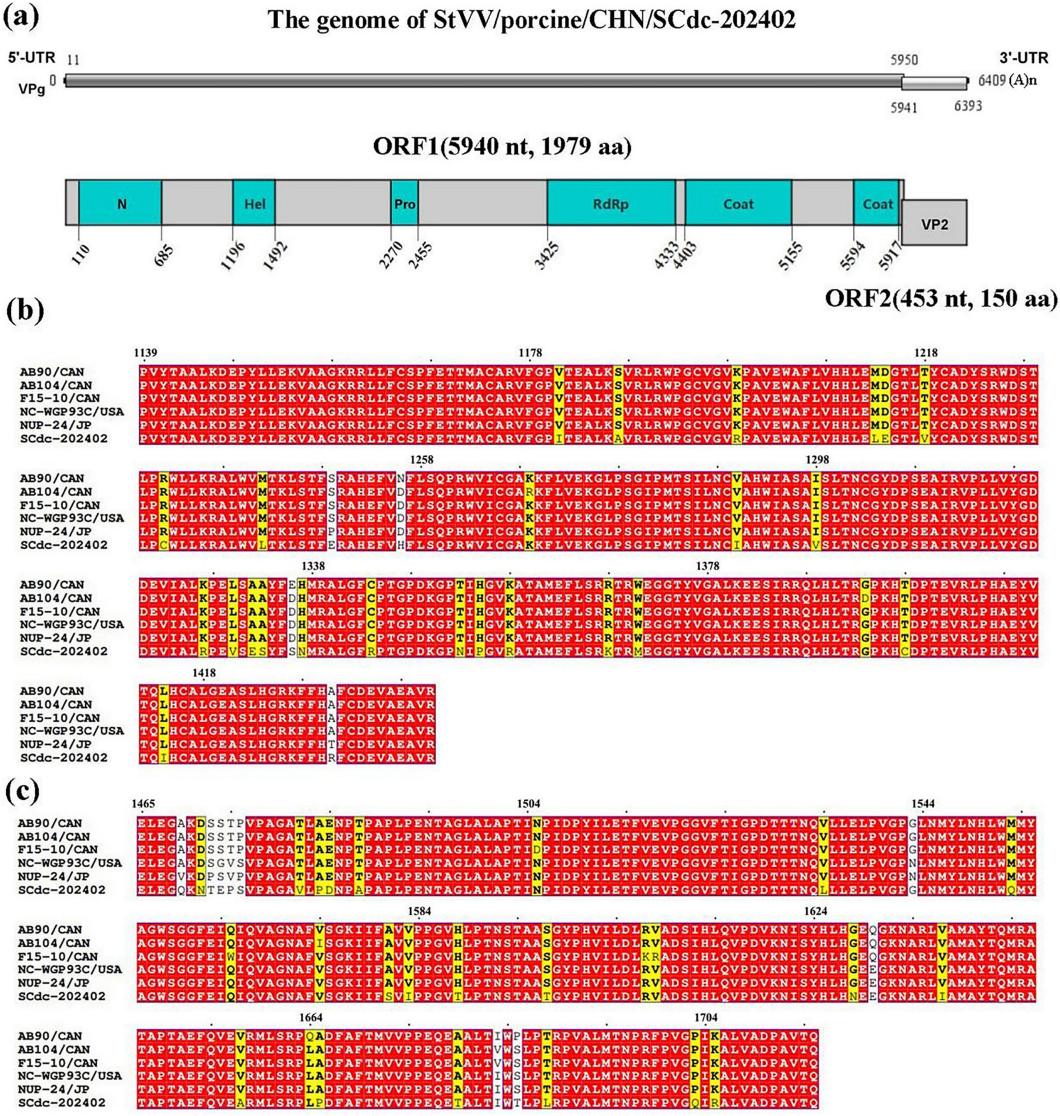
Schematic representation of the putative genomic organization and phylogenetic tree based on full-length genomic sequence of StVV/porcine/CHN/SCdc-202402. **(a)** N, Viral polyprotein N-terminal; Hel, RNA helicase; Pro, Southampton virus-type processing protease; RdRP, ps-ssRNAv RdRp-like; coat, calicivirus coat protein. **(b)** Location of the mutation site of the SCdc-202402 strain in the RdRp. The unique amino acids are indicated with highlighted boxes. **(c)** Location of the mutation site of the StVV/porcine/CHN/SCdc-202402 strain in the Coat1. The unique amino acids are indicated with highlighted boxes.

The genomes of *Caliciviridae* are approximately 6.4–8.5 kilobases in length and contain 2–3 non-overlapping ORFs, varying by genus (25). In this study, the complete nucleotide sequence of a StVV strain was determined and named StVV/porcine/CHN/SCdc-202402 (GenBank Accession No. PV740161). The genome length of SCdc-202402 was 6,409 bp, with 54.97% GC content. Two putative ORFs (ORF1 and 2) were identified within the genome of StVV/porcine/CHN/SCdc-202402. The 5′ proximal ORF1, starting at 11 nt and ending at 5,950 nt (5,940 nt long) encoded a predicted protein of 1,979 aa, including non-structural proteins and structural proteins. A search for conserved domains showed that the ORF1-encoded protein contains the viral polyprotein N-terminal (N), RNA helicase (Hel), Southampton virus-type processing protease (Pro), ps-ssRNAv RdRp-like (RdRp), and two Calicivirus coat proteins (Coat 1 and Coat 2) (Figure 4a). The 3′ proximal ORF2 starts at 5,941 nt and ends at 6,393 nt (453 nt long) encoded a predicted structural protein of 150 aa (VP2). In addition, VPg is covalently attached to the 5′-end, and a polyadenylated tail is present in the 3′-terminus of the genome (Figure 4a). The results indicated that the SCdc-202402 strain has a typical genome structure of *Caliciviridae* (29).

Amino acid mutation analysis showed that RdRp and Coat1 are the most variable regions in the nonstructural proteins and structural proteins in the genome of StVV/porcine/CHN/SCdc-202402. In the strain, a total of 27 unique amino acids, such as I1181, A1187, R1199, L1213, E1214, V1218, C1232, L1242, E1249, H1256, I1290, V1298, R1327, V1330, E1332, S1333, S1336, N1337, R1344, N1353, P1355, R1358, K1368, M1371, C1398, I1414, and R1431, were identified in the *RdRp* gene (Figure 4b), and 24 unique amino acids, such as Q1469, N1471, T1472, E1473, P1474, V1481, P1483, D1484, A1487, L1534, Q1553, S1581, I1583, T1588, T1597, N1628, I1637, A1657, P1665, T1679, T1685, L1688, Q1703, and R1705, were identified in the *Coat1* gene when compared to the five representative swine-origin strains (Figure 4c).

**Figure 5:**
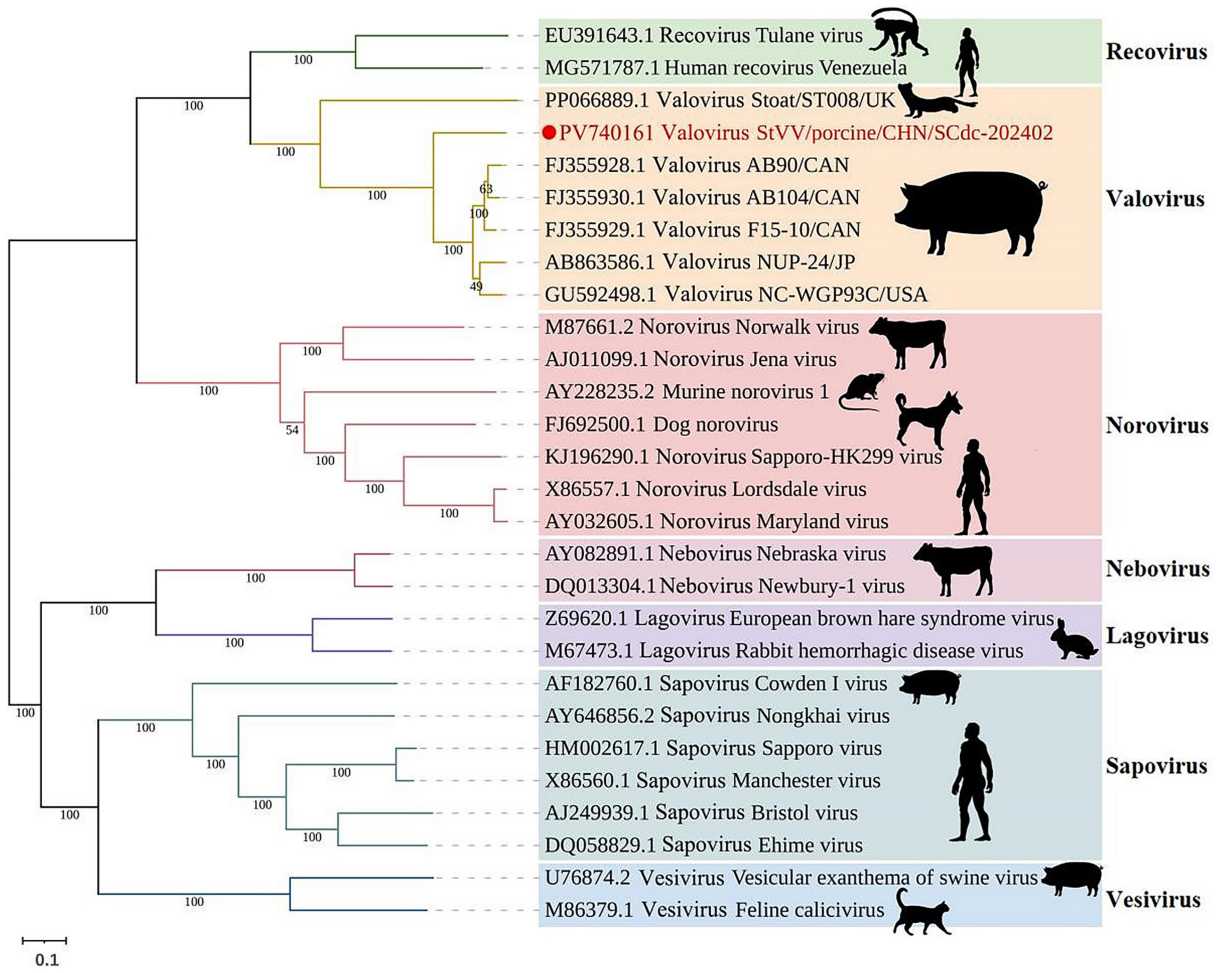
Schematic representation of the phylogenetic tree based on full-length genomic sequence of StVV/porcine/CHN/SCdc-202402. Maximum likelihood (ML) phylogenetic trees were inferred using a whole genome sequences, the phylogenetic trees were constructed with 1,000 replicates using the MEGA11 program. The scale bar indicates evolutionary distance in numbers of substitutions per amino acid site.

Family *Caliciviridae* is composed of 11 established genera, including 7 genera (*Lagovirus, Norovirus, Nebovirus, Recovirus, Sapovirus, Valovirus,* and *Vesivirus*) from mammals, 2 genera (*Bavovirus* and *Nacovirus*) from birds, and 2 genera (*Minovirus* and *Salovirus*) from fish (42–44), indicating that caliciviruses have a broad viral infection spectrum. In phylogenetic analysis, all mammalian *Caliciviridae* strains are separated into seven phylogenetic clades: *Vesivirus*, *Recovirus*, *Lagovirus*, *Nebovirus*, *Sapovirus*, *Valovirus* and *Norovirus*. In swine, StVV strains detected to date have been classified into 3 genera: *Valovirus*, *Sapovirus*, and *Vesivirus*. The StVV/porcine/CHN/SCdc-202402 strain separated into genus *Valovirus*, together with five strains (AB90, AB104, F15-10, NUP-24, and NC-WGP93C) from pigs and one strain (ST008) from stoat. Interestingly, the StVV/porcine/CHN/SCdc-202402 was classified into a single evolutionary branch with bootstrap value of 100, and showed a distant genetic distance of 0.2862–0.2940 with the five swine-origin StVV strains from Canada, U.S.A., and Japan (distance among five strains arranged from 0.0556 to 0.1174) (Figure 5; Supplementary Table S3), suggesting that StVV/porcine/CHN/SCdc-202402 have undergone distinct evolution process in China and may represent a new species within the genus *valovirus*.

Genomic sequence comparison and analysis showed the genome of StVV/porcine/CHN/SCdc-202402 shared the highest nucleotide identity (82.6%) with a *Valovirus* strain NUP-24 from Japan, which was identified in fecal samples from swine in 2013 (28). However, the genome of StVV/porcine/CHN/SCdc-202402 showed lower identity (<60%), in terms of 26.1–51.3%, compared to reference genomes from other 6 genera. The ORF1 and ORF2 of StVV/porcine/CHN/SCdc-202402 shared 81.0–95.0% nucleotide (67.6–92.1% aa) sequence identity with swine-origin *Valovirus* strains, a significantly higher percentage than with the other reference strains from *Lagovirus* (29.4–84.8% nt, 14.9–82.2% aa), *Nebovirus* (31.7–75.7% nt, 17.3–71.4% aa), *Recovirus* (31.0–45.7% nt, 30.2–35.0% aa), *Vesivirus* (8.2–27.1% nt, 8.9–17.4% aa), *Norovirus* (30.1–35.8% nt, 20.2–33.8% aa), and *Sapovirus* (30.3–83.7% nt, 15.7–80.4% aa) (Table 2). The data in the present study show that the StVV/porcine/CHN/SCdc-202402 from Tibetan pigs is closely related to StVV strains that originated from domestic pigs.

**Table 2:** Nucleotide and amino acid sequence identity values for different regions compared with other *Caliciviridae* reference strains.

Genus	Species	Accession no.	ORF1 (nt/aa)	ORF2 (nt/aa)	Complete genome (nt)
*Lagovirus*	European brown hare	Z69620.1	29.4/14.9	84.7/82.2	35.1
	Rabbit	M67473.1	29.4/14.9	84.8/82.0	34.4
*Nebovirus*	Bovine Bovine	AY082891.1	31.7/17.5	75.5/71.4	33.9
		DQ013304.1	31.9/17.3	75.7/71.4	34.0
*Recovirus*	Monkey	EU391643.1	44.6/34.5	31.8/32.1	51.3
	*Homo sapiens*	MG571787.1	45.7/35.0	31.0/30.2	51.3
*Vesivirus*	Swine	U76874.2	23.5/13.4	8.2/8.9	26.1
	Feline	M86379.1	27.1/17.4	13.6/15.2	31.3
*Norovirus*	*Homo sapiens*	M87661.2	31.3/20.2	33.2/33.3	38.4
	*Canis lupus familiaris*	FJ692500.1	35.6/24.1	33.7/33.8	39.0
	Bovine	AJ011099.1	34.9/24.6	35.1/33.8	41.7
	*Homo sapiens*	X86557.1	35.8/24.8	31.9/32.0	41.0
	*Homo sapiens*	AY032605.1	35.6/24.2	31.9/31.9	40.8
	Murine	AY228235.2	34.8/24.3	32.1/30.5	42.0
	*Homo sapiens*	KJ196290.1	35.1/23.6	30.1/29.3	40.3
*Sapovirus*	*Homo sapiens*	HM002617.1	30.7/15.8	83.4/80.2	35.2
		AJ249939.1	31.0/15.8	82.9/79.7	35.2
		DQ058829.1	31.3/16.3	83.3/79.8	35.5
		X86560.1	30.5/16.2	83.5/80.1	35.2
		AY646856.2	30.6/15.7	83.7/80.1	34.6
	Swine	AF182760.1	30.3/16.9	83.6/80.4	36.0
*Valovirus*	Stoat	PP066889.1	58.5/42.6	86.1/82.9	61.0
	Swine	AB863586.1	81.4/67.7	**95.0/92.1**	**82.6**
		FJ355928.1	81.0/67.6	94.7/91.7	81.9
		FJ355929.1	81.3/**68.5**	94.6/91.8	82.2
		FJ355930.1	**81.5/**68.1	94.6/91.6	82.3
		GU592498.1	81.3/68.3	94.5/91.7	82.3
		HM014307	NA	94.7/91.8	NA

The highest nucleotide and amino acid identities are indicated in bold typeface.

### 3.4 Virus detection rate in fecal samples from diarrheic cases versus controls

To confirm the metagenomic results and to determine the viral prevalence of DCV and StVV in individual samples from Tibetan pigs, 27 diarrheic samples and 60 asymptomatic fecal samples were subjected to specific RT-PCR amplification and sequencing. The results showed the detection of 21 DCV-positive and 2 StVV-positive strains of the 27 diarrheic samples with a positive rate of 77.8% (21/27) and 7.4% (2/27), respectively, but they were both negative in all the 60 asymptomatic fecal samples. The resulting *p* value was significant at < 0.0001 for DCV, while nonsignificant at 0.094 for StVV (Table 3). Interestingly, the 2 StVV-positive samples were detected positive for DCV with a co-infection rate of 7.4% (2/27), however, the resulting *p* value was nonsignificant at 0.094 (Table 3). The results suggesting the DCV might be associated with diarrhea in Tibetan pigs. However, owing to the limited number of fecal samples collected from a single pig farm, the detection data above cannot represent the prevalence of DCV and StVV in Tibetan pigs. Therefore, a larger number of clinical samples, including sera, rectal, or nasal swab samples, from different counties in Ganzi Tibetan autonomous prefecture will be collected in future studies. Co-infections with multiple viruses were common in the pigs with diarrhoeal disease (7), whether single infections with DCV or StVV, or co-infections with them both, or specific combinations of viruses are more likely to result in diarrhoeal disease in Tibetan pigs will require collecting larger number of cases and controls, and analyzing more viruses, such as rotavirus, torovirus, and astrovirus, etc. (Figure 1).

**Table 3:** Virus detection rates in diarrheic samples versus controls.

Virus	Family	Genus	No. positive/total (% positive)		*p*-value ^a^
			Asymptomatic samples	Samples with diarrhea	
DCV	*Dicistroviridae*	unassigned	0/60 (0%)	21/27 (77.8%)	<0.0001^*^
StVV	*Caliciviridae*	*Valovirus*	0/60 (0%)	2/27 (7.4%)	0.094
DCV + StVV	/	/	0/60 (0%)	2/27 (7.4%)	0.094

a *, significant.

Viral diarrhea is a common disease in Tibetan pigs that causing high mortality in suckling piglets. Using high-throughput sequencing and metagenomic analysis, several viruses that can cause diarrhoeal disease in these animals have been identified, including porcine epidemic diarrhea virus (PEDV) (3), porcine deltacoronavirus (PDCoV) (6), porcine bocavirus (PBoV) (45), porcine kobuvirus (PKoV) (36), sapelovirus (33), and porcine circovirus 2 (PCV-2) (46). In this study, two novel viruses (DCV and StVV) have been identified in Tibetan pigs with porcine diarrheal disease in Daocheng County (altitude ~4,000 m). For virus isolation, the fecal suspensions of DCV-positive and StVV-positive samples were passed through 0.22-μm filters and inoculated onto Vero and PK-15 cell lines. However, after blindly passaged in cells for five passages, no visible CPE was observed and RT-PCR detecting was negative for DCV and StVV. Several potential reasons were considered for the failure to isolate the two novel viruses: (i) the Vero/PK-15 cell line has low susceptibility to DCV and StVV; (ii) the two enteric viruses may have a strong tissue tropism for porcine intestinal cell lines or primary cells; (iii) during the process of virus isolation, there may be a lack of certain proteases, such as trypsin (47). Therefore, with the gradual progress of the experiment, we attempt to use other cell lines (such as porcine intestinal epithelial cells, IPEC-J2) or intestinal organoid cultures for viral isolation. Meanwhile, the impact of different concentrations of trypsin on the virus isolation of DCV and StVV will be evaluated in the future studies.

Although DCV have been detected in vertebrate and invertebrate animals, the pathogenicity of DCV strains from different hosts varied greatly (8). The DCV strains from invertebrates have been reported highly pathogenic to their hosts, such as the Israeli acute paralysis virus (IAPV) infecting honeybees and Taura syndrome virus (TSV) in shrimps, leading to a high mortality (17, 18). However, the symptoms of DCV infection in vertebrate animals and human are mainly manifested as asymptomatic or diarrhea (19–23). Due to all DCV sequences detected in animals were based on metaviromic analyses, it still remains to be determined whether the virus is capable of replication in the intestinal tract of swine, and the clinical significance caused by DCV in vertebrate animals and human remains to be explored.

## 4 Conclusion

In conclusion, two novel viruses (DCV/porcine/CHN/SCdc-2024 and StVV/porcine/CHN/SCdc-202402) were identified in Tibetan pigs with porcine diarrheal disease on the Tibetan plateau of southwest China. The novel dicistrovirus was found for the first time in swine host and may represent members of a potential new genera within the *Dicistroviridae* family. Additionally, a StVV strain was identified in China for the first time and showed unique phylogenetic branch compared with other swine-origin StVV strains. Interestingly, the DCV was significantly associated with diarrhea in Tibetan pigs. However, detection of the DCV in the pig feces is likely to indicate a dietary origin for the virus. Whether the virus contributed to the development of diarrhea remains to be assessed in future studies.

## Data Availability

The datasets presented in this study can be found in online repositories. The names of the repository/repositories and accession number(s) can be found in the article/Supplementary material.

## Footnotes

^1^ https://github.com/OpenGene/fastp

^2^ https://github.com/ablab/spades

^3^ https://www.ncbi.nlm.nih.gov/orffinder/

^4^ https://www.ncbi.nlm.nih.gov/structure/cdd/wrpsb.cgi

^5^ https://ictv.global/report/chapter/dicistroviridae/dicistroviridae

^6^ https://ictv.global/report/chapter/caliciviridae
