# Supplementary material for: Emergence of two novel viruses in Tibetan pigs with porcine diarrheal disease on the Tibetan plateau of southwest China
Source: Front Vet Sci. 2025 Sep 3;12:1654388. doi: 10.3389/fvets.2025.1654388 (PMC12440765; doi:10.3389/fvets.2025.1654388)
Supplement: Supplementary file 1 [file Table_1.DOCX]

Supplementary Material

# Supplementary Tables

## Supplementary Tables

**Supplementary Table 1.** Specific primers used to amplify the genome of DCV/porcine/CHN/SCdc-2024 in this study.

| Fragment | Primer name ^a^ | Primers sequence (5´-3´) | Primer position | Length (bp) |
| --- | --- | --- | --- | --- |
| 1 | 1F | CGGAGGGGCATCTTTTCAC | 1-19 | 1847 |
|  | 1R | CCTCCAGAAGCGACATCA | 1830-1847 |  |
| 2 | 2F | CTTTAGTGATTCTGGCTTCT | 1563-1583 | 1959 |
|  | 2R | TCAATTTCATCTCCGTTG | 3504-3521 |  |
| 3 | 3F | TCCGAGGATAAGGAGAAG | 3376-3397 | 1986 |
|  | 3R | AGACCTGGATTACTGTTGA | 5344-5361 |  |
| 4 | 4F | TCGCAAGAATGTGCTGAC | 5248-5260 | 1872 |
|  | 4R | AAGGGATGATAGAAACATAAGG | 7098-7119 |  |
| 5 | 5F | ATGATAACACGCTTTACACCT | 6912-6929 | 1400 |
|  | 5R | GCTGTAAAATAGCTGTTTCTTAAAG | 8287-8311 |  |

^a^ F and R represent forward and reverse primers, respectively.

**Supplementary Table 2.** Specific primers used to amplify the genome of StVV/porcine/CHN/SCdc-202402 in this study.

| Fragment | Primer name ^a^ | Primers sequence (5´-3´) | Primer position | Length (bp) |
| --- | --- | --- | --- | --- |
| 1 | 1F | TTGAACTGCCATGTCTGTCG | 1-20 | 1324 |
|  | 1R | GTAGGAGTCCCAATGGTCGAT | 1304-1324 |  |
| 2 | 2F | GTGCAGAGGCATCTTTGGCGC | 1125-1145 | 1272 |
|  | 2R | CACCACGTTTCTTGGCGTCAG | 2376-2396 |  |
| 3 | 3F | AACAGGCCCAACGTAGCCTTC | 2048-2068 | 1395 |
|  | 3R | GGCTGCCGTGTAGACAGGTTGA | 3421-3442 |  |
| 4 | 4F | TGGCCCTGACTGGACTCGA | 3153-3171 | 1293 |
|  | 4R | CAGCTGGAACAGAGGGTTCA | 4426-4445 |  |
| 5 | 5F | GCACTCGGTGAGGCCTCCCT | 4259-4278 | 1220 |
|  | 5R | AGGGCGGCTTGCGCTGTGAT | 5459-5478 |  |
| 6 | 6F | ACACCAGATCGAGATGATGA | 5266-5285 | 1144 |
|  | 6R | AATGAGTTTTCAAAGCTCAG | 6390-6409 |  |

^a^ F and R represent forward and reverse primers, respectively.

**Supplementary Table 3.** Pairwise compare of genetic distance values among Caliciviridae strains.

|  | **Z69620.1_European_brown_hare_syndrome_virus** | **M67473.1_Rabbit_hemorrhagic_disease_virus** | **AY082891.1_Nebraska_virus** | **DQ013304.1_Newbury-1_virus** | **M87661.2_Norwalk_virus** | **FJ692500.1_Dog_norovirus** | **AJ011099.1_Jena_virus** | **X86557.1_Lordsdale_virus** | **AY032605.1_Maryland_virus** | **AY228235.2_Murine_norovirus_1** | **KJ196290.1_Sapporo-HK299_virus** | **EU391643.1_Tulane_virus** | **MG571787.1_Human_recovirus_Venezuela** | **HM002617.1_Sapporo_virus** | **AJ249939.1_Bristol_virus** | **AF182760.1_Cowden_I_virus** | **DQ058829.1_Ehime_virus** | **X86560.1_Manchester_virus** | **AY646856.2_Nongkhai_virus** | **U76874.2_Vesicular_exanthema_of_swine_virus** | **M86379.1_Feline_calicivirus** | **AB863586.1_NUP-24/JP** | **FJ355928.1_AB90/CAN** | **FJ355929.1_F15-10/CAN** | **FJ355930.1_AB104/CAN** | **GU592498.1_NC-WGP93C/USA** | **PP066889.1_Stoat/ST008/UK** | **StVV/porcine/CHN/SCdc-2024** |
| --- | --- | --- | --- | --- | --- | --- | --- | --- | --- | --- | --- | --- | --- | --- | --- | --- | --- | --- | --- | --- | --- | --- | --- | --- | --- | --- | --- | --- |
| **Z69620.1_European_brown_hare_syndrome_virus** |  |  |  |  |  |  |  |  |  |  |  |  |  |  |  |  |  |  |  |  |  |  |  |  |  |  |  |  |
| **M67473.1_Rabbit_hemorrhagic_disease_virus** | 0.3580 |  |  |  |  |  |  |  |  |  |  |  |  |  |  |  |  |  |  |  |  |  |  |  |  |  |  |  |
| **AY082891.1_Nebraska_virus** | 1.0231 | 1.0083 |  |  |  |  |  |  |  |  |  |  |  |  |  |  |  |  |  |  |  |  |  |  |  |  |  |  |
| **DQ013304.1_Newbury-1_virus** | 1.0146 | 1.0169 | 0.1657 |  |  |  |  |  |  |  |  |  |  |  |  |  |  |  |  |  |  |  |  |  |  |  |  |  |
| **M87661.2_Norwalk_virus** | 1.9071 | 1.9900 | 1.7957 | 1.7740 |  |  |  |  |  |  |  |  |  |  |  |  |  |  |  |  |  |  |  |  |  |  |  |  |
| **FJ692500.1_Dog_norovirus** | 1.8314 | 1.8561 | 1.6995 | 1.7172 | 0.7529 |  |  |  |  |  |  |  |  |  |  |  |  |  |  |  |  |  |  |  |  |  |  |  |
| **AJ011099.1_Jena_virus** | 1.8560 | 1.8287 | 1.7551 | 1.6837 | 0.5644 | 0.7500 |  |  |  |  |  |  |  |  |  |  |  |  |  |  |  |  |  |  |  |  |  |  |
| **X86557.1_Lordsdale_virus** | 1.8419 | 1.8194 | 1.8234 | 1.7937 | 0.7659 | 0.5980 | 0.7914 |  |  |  |  |  |  |  |  |  |  |  |  |  |  |  |  |  |  |  |  |  |
| **AY032605.1_Maryland_virus** | 1.8677 | 1.8624 | 1.8270 | 1.7950 | 0.7710 | 0.5929 | 0.7954 | 0.0576 |  |  |  |  |  |  |  |  |  |  |  |  |  |  |  |  |  |  |  |  |
| **AY228235.2_Murine_norovirus_1** | 1.9284 | 1.8825 | 1.7613 | 1.7423 | 0.8248 | 0.7422 | 0.8231 | 0.7485 | 0.7529 |  |  |  |  |  |  |  |  |  |  |  |  |  |  |  |  |  |  |  |
| **KJ196290.1_Sapporo-HK299_virus** | 1.8728 | 1.8252 | 1.7503 | 1.7631 | 0.7233 | 0.6075 | 0.7708 | 0.4373 | 0.4403 | 0.7721 |  |  |  |  |  |  |  |  |  |  |  |  |  |  |  |  |  |  |
| **EU391643.1_Tulane_virus** | 1.7628 | 1.8092 | 1.8727 | 1.8796 | 1.2842 | 1.3473 | 1.3614 | 1.3382 | 1.3409 | 1.3715 | 1.3257 |  |  |  |  |  |  |  |  |  |  |  |  |  |  |  |  |  |
| **MG571787.1_Human_recovirus_Venezuela** | 1.8050 | 1.8273 | 1.8303 | 1.8368 | 1.2943 | 1.3936 | 1.3397 | 1.3287 | 1.3415 | 1.3717 | 1.2984 | 0.6303 |  |  |  |  |  |  |  |  |  |  |  |  |  |  |  |  |
| **HM002617.1_Sapporo_virus** | 1.4110 | 1.3886 | 1.4121 | 1.4106 | 1.7522 | 1.7093 | 1.7438 | 1.6718 | 1.6673 | 1.7670 | 1.6589 | 1.8230 | 1.7631 |  |  |  |  |  |  |  |  |  |  |  |  |  |  |  |
| **AJ249939.1_Bristol_virus** | 1.3866 | 1.4147 | 1.3800 | 1.3811 | 1.8192 | 1.6368 | 1.7943 | 1.7031 | 1.6976 | 1.7170 | 1.6983 | 1.8172 | 1.8068 | 0.5654 |  |  |  |  |  |  |  |  |  |  |  |  |  |  |
| **AF182760.1_Cowden_I_virus** | 1.4150 | 1.4548 | 1.4260 | 1.4280 | 1.7869 | 1.6957 | 1.7726 | 1.7280 | 1.7223 | 1.6765 | 1.7675 | 1.8128 | 1.8027 | 0.8838 | 0.8370 |  |  |  |  |  |  |  |  |  |  |  |  |  |
| **DQ058829.1_Ehime_virus** | 1.4445 | 1.4288 | 1.3704 | 1.3719 | 1.7934 | 1.7344 | 1.7190 | 1.7296 | 1.7285 | 1.6749 | 1.7333 | 1.8824 | 1.7880 | 0.5613 | 0.4151 | 0.8505 |  |  |  |  |  |  |  |  |  |  |  |  |
| **X86560.1_Manchester_virus** | 1.4238 | 1.3984 | 1.3798 | 1.4147 | 1.7671 | 1.7205 | 1.7568 | 1.6702 | 1.6706 | 1.7333 | 1.6467 | 1.8281 | 1.8120 | 0.0861 | 0.5719 | 0.8849 | 0.5493 |  |  |  |  |  |  |  |  |  |  |  |
| **AY646856.2_Nongkhai_virus** | 1.3586 | 1.4032 | 1.3740 | 1.3871 | 1.9220 | 1.8423 | 1.8442 | 1.7681 | 1.7604 | 1.8101 | 1.7669 | 1.8982 | 1.8611 | 0.6945 | 0.7269 | 0.8692 | 0.6975 | 0.6860 |  |  |  |  |  |  |  |  |  |  |
| **U76874.2_Vesicular_exanthema_of_swine_virus** | 1.4488 | 1.4488 | 1.5414 | 1.5437 | 2.0090 | 1.8966 | 1.9652 | 1.8835 | 1.8942 | 1.9642 | 1.9392 | 2.0900 | 1.9868 | 1.2671 | 1.2636 | 1.3213 | 1.2788 | 1.2694 | 1.2654 |  |  |  |  |  |  |  |  |  |
| **M86379.1_Feline_calicivirus** | 1.4498 | 1.4728 | 1.4962 | 1.5233 | 1.9197 | 1.8992 | 2.0417 | 1.7982 | 1.8021 | 1.9242 | 1.8601 | 2.0878 | 1.9125 | 1.2422 | 1.2466 | 1.3545 | 1.2688 | 1.2418 | 1.2513 | 0.6319 |  |  |  |  |  |  |  |  |
| **AB863586.1_NUP-24/JP** | 1.6544 | 1.7132 | 1.8000 | 1.7905 | 1.3055 | 1.2264 | 1.2306 | 1.2360 | 1.2426 | 1.2576 | 1.2708 | 0.9990 | 0.9754 | 1.6897 | 1.6279 | 1.6794 | 1.6940 | 1.6767 | 1.6999 | 1.8918 | 1.9934 |  |  |  |  |  |  |  |
| **FJ355928.1_AB90/CAN** | 1.6532 | 1.6849 | 1.7620 | 1.7567 | 1.3015 | 1.2454 | 1.2448 | 1.2692 | 1.2828 | 1.2655 | 1.2682 | 1.0066 | 0.9694 | 1.6780 | 1.6339 | 1.6496 | 1.6887 | 1.6713 | 1.7220 | 1.9382 | 1.8962 | 0.1156 |  |  |  |  |  |  |
| **FJ355929.1_F15-10/CAN** | 1.6577 | 1.7028 | 1.7826 | 1.7548 | 1.3069 | 1.2450 | 1.2346 | 1.2515 | 1.2643 | 1.2714 | 1.2702 | 0.9957 | 0.9563 | 1.6943 | 1.6518 | 1.6763 | 1.6875 | 1.6802 | 1.7251 | 1.9103 | 1.9345 | 0.1174 | 0.0604 |  |  |  |  |  |
| **FJ355930.1_AB104/CAN** | 1.6608 | 1.7277 | 1.7959 | 1.7866 | 1.3158 | 1.2488 | 1.2585 | 1.2639 | 1.2775 | 1.2685 | 1.2700 | 1.0053 | 0.9749 | 1.6796 | 1.6557 | 1.6567 | 1.6869 | 1.6621 | 1.7055 | 1.9434 | 1.9213 | 0.1153 | 0.0556 | 0.0581 |  |  |  |  |
| **GU592498.1_NC-WGP93C/USA** | 1.6809 | 1.7201 | 1.7792 | 1.7911 | 1.3026 | 1.2316 | 1.2564 | 1.2568 | 1.2613 | 1.2745 | 1.2465 | 0.9953 | 0.9731 | 1.7063 | 1.6568 | 1.6756 | 1.7096 | 1.6733 | 1.6869 | 1.8977 | 1.9508 | 0.1078 | 0.1142 | 0.1112 | 0.1098 |  |  |  |
| **PP066889.1_Stoat/ST008/UK** | 1.9639 | 1.9700 | 1.9288 | 1.9324 | 1.3595 | 1.2845 | 1.3627 | 1.3729 | 1.3680 | 1.3780 | 1.3555 | 1.0943 | 1.0403 | 1.7432 | 1.7552 | 1.9241 | 1.7363 | 1.7199 | 1.8254 | 1.9594 | 1.9930 | 0.7803 | 0.7847 | 0.7946 | 0.7926 | 0.7904 |  |  |
| **StVV/porcine/CHN/SCdc-2024** | 1.7438 | 1.8178 | 1.8345 | 1.8138 | 1.3152 | 1.2761 | 1.2866 | 1.2694 | 1.2802 | 1.3126 | 1.3115 | 1.0455 | 1.0042 | 1.7821 | 1.7381 | 1.6933 | 1.6945 | 1.7785 | 1.7511 | 1.9423 | 2.0013 | 0.2862 | 0.2940 | 0.2886 | 0.2871 | 0.2905 | 0.8213 |  |
